# “Osteolipoma of buccal mucosa: Case report and literature review”

**DOI:** 10.4317/jced.52803

**Published:** 2016-04-01

**Authors:** Jayam Raviraj, Vijay Kumar-Bokkasam, Dirasantchu Suresh, Suman Venkata

**Affiliations:** 1 MDS, DNB, Professor, Dept. of Oral Medicine and Radiology, CKS Theja Institute of Dental Sciences & Research, Tirupati-A.P, India; 2 Professor, Dept. of Oral Medicine and Radiology, CKS Theja Institute of Dental Sciences & Research, Tirupati-A.P, India; 3 Senior Lecturer, Dept. of Oral Medicine and Radiology, CKS Theja Institute of Dental Sciences & Research, Tirupati-A.P, India; 4 Reader, Dept. of Oral Medicine and Radiology, CKS Theja Institute of Dental Sciences & Research, Tirupati-A.P, India

## Abstract

Osteolipoma affecting oral cavity is indeed rare. We hereby report a case of osteolipoma affecting buccal mucosa. A review of literature of osteolipoma of oral cavity, particularly on radiographic/imaging findings was done. Only 16 cases of Osteolipoma of oral cavity are reported in the literature. The radiographic findings of our case, i.e. multiple dense homogenous radio-opaque structures was reported earlier only in one case [out of 16] of osteolipoma of oral cavity.

** Key words:**Lipoma, osteolipoma, panoramic radiography, radio-opaque, radiography.

## Introduction

Lipoma is a benign tumor composed of mature fat tissue arranged in lobules that are separated by fibrous septa surrounded by their fibrous capsule. Lipoma can affect any part of the body, with only 1-4% of them affecting oral cavity. Buccal mucosa, floor of the mouth, tongue and lip are the most common sites affected (1). Histological variants of lipoma include: spindle cell lipoma, fibrolipoma, myolipoma, myxolipoma, angiolipoma, osteolipoma and chondrolipoma. Osteolipomas are less common than chondrolipomas and normally are presented in large and long term evolution lesions (2).

On reviewing the English literature, only 16 cases of osteolipoma affecting oral cavity were reported. Osteolipoma affecting para-oral structures like parotid gland, submandibular space, parapharyngeal space etc. were excluded and only cases affecting the oral cavity were included for reviewing the radiographic/imaging findings, particularly. Out of them, only seven cases have described the radiographic/imaging findings of Osteolipoma affecting oral cavity. Moreover, none of the cases had radiographic features which were similar to our case, except for one case, which includes the presence of multiple homogenous dense radio-opaque structures. The site of the occurrence of the present lesion, which is retro-commissural area, is also unique and not been reported earlier.

We hereby report a case of osteolipoma of the left retro-commissural area with a unique radiographic presentation.

## Case Report

**Figure 1:**
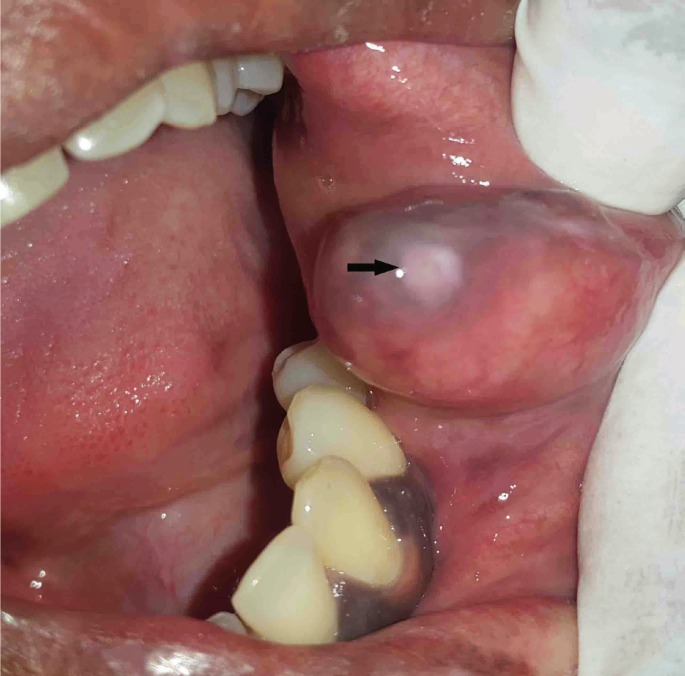
Showing swelling in the left buccal mucosa with white surface discoloration [black arrow].

A 38 year old female patient presented with a chief complaint of painless swelling in her left inner cheek region since 28 years, which first appeared as a small nodule and gradually increased to attain the present size. On intra-oral examination, a solitary swelling located at the left retro-commisural area, measuring approx. 2x2x3 cms, the surface of which exhibited a small white discoloration which is perhaps secondary to surface trauma (Fig. 1). On palpation, the swelling was non-tender, fluctuant, soft in consistency although a few hard globular structures could be palpated suggestive of probable calcified structures. A clinical differential diagnosis of long standing lipoma, mucocele and benign minor salivary gland tumor was considered.

**Figure 2:**
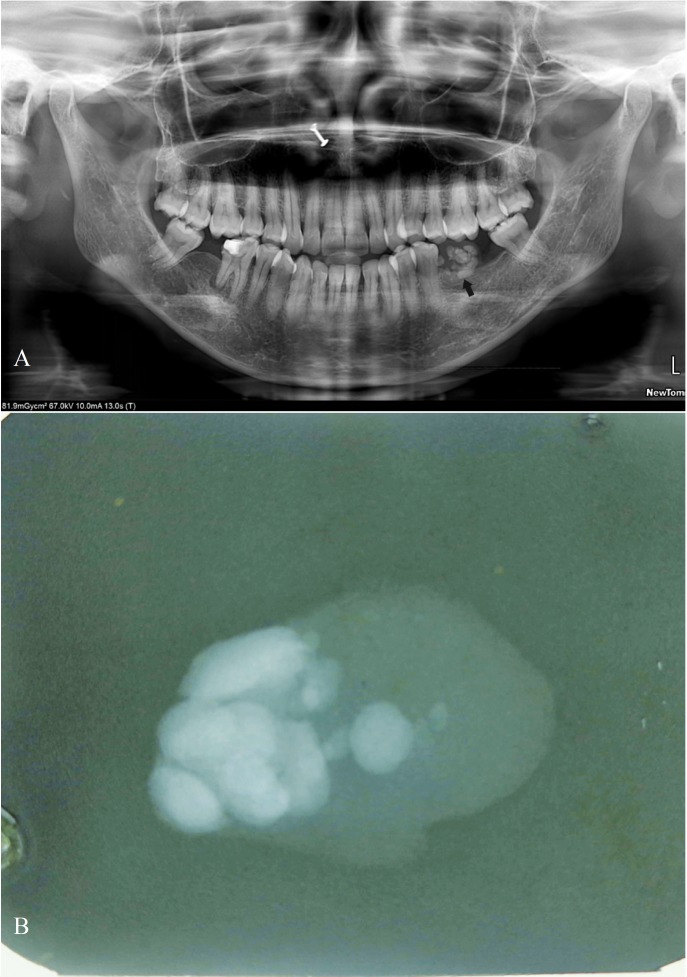
A) Panoramic radiograph revealing multiple dense homogenous radio-opacities in the left mandibular posterior region [black arrow]. B) Radiograph of resected specimen showing multiple dense homogenous radio-opacities of varying size and shapes.

**Figure 3:**
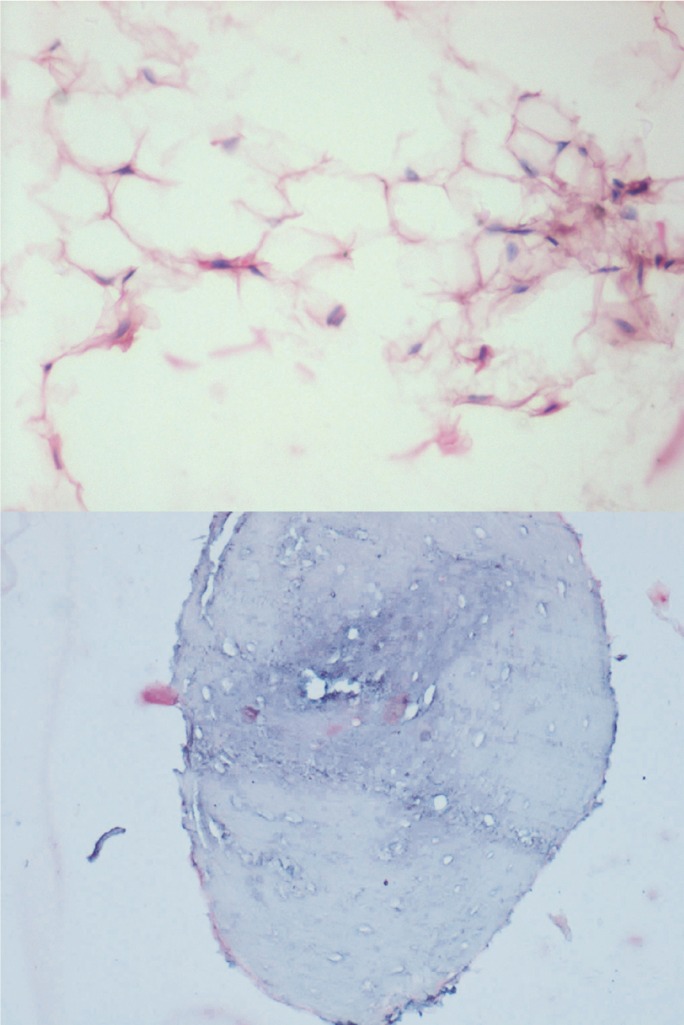
A) Histopathological pictures showing adipose tissue and bony trabeculae [H&E, x25]. B) Histopathological pictures showing adipose tissue and bony trabeculae [H&E, x25].

Panoramic radiograph (Fig. 2A) revealed multiple dense homogenous radio-opaque structures of varying sizes and shapes in the left mandibular posterior region [edentulous region of 36 & 37]. These radio-opaque structures were presumed to be super-imposition of soft tissue lesional calcifications as they corresponded with the anatomic site of the lesion and also with the palpatory findings of hard globular structures. An excisional biopsy was performed under local anesthesia and the resected specimen was subjected to radiography using an intra-oral film, which again revealed multiple dense homogenous radio-opaque calcified structures (Fig. 2B). The same specimen was later submitted for histopathological examination with H&E staining which revealed lesional tissue comprising predominantly of adipose tissue along with intervening fibrous connective tissue, few inflammatory cells and thin walled blood vessels (Fig. 3A). Decalcified sections showed bony trabeculae with embedded osteocytes, some of the trabeculae showed empty lacunae (Fig. 3B).

Based on clinico-radiological and histopathological correlation, a final diagnosis of Osteolipoma was considered.

## Discussion

Lipomas containing osseous tissue are either intra-osseous or parosteal variety (usually attached to trunk bones). However pure soft tissue osteolipomas are rare. To our knowledge only 16 cases of intra-oral osteolipomas are reported. The age/sex, site, clini-cal presentation, duration of the lesion and radiographic presentation of these cases are described in  Table 1.

**Table 1:**
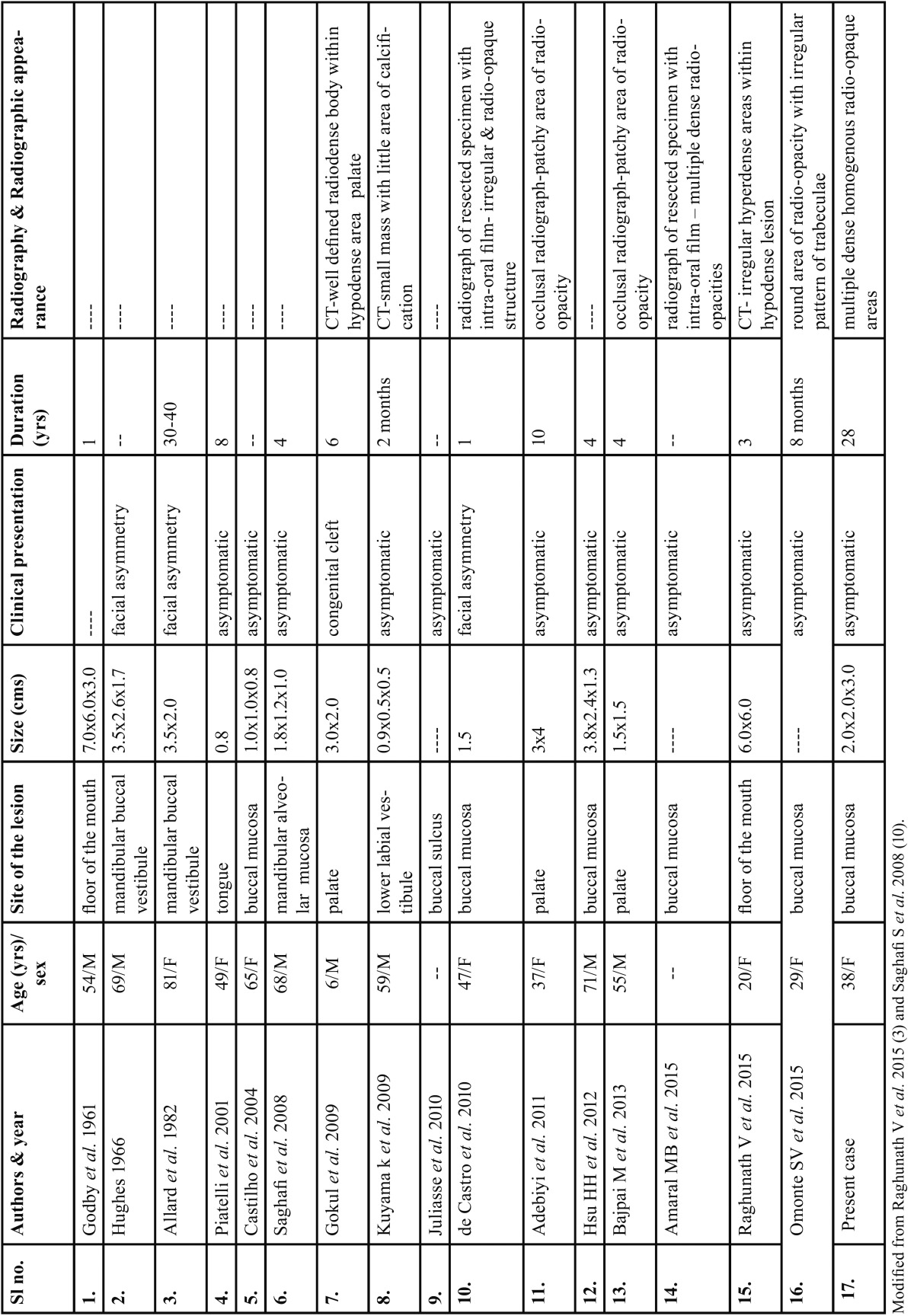
Previously reported cases of osteolipoma of oral cavity with radiographic/imaging findings.

On reviewing the literature the age of oral osteolipoma patients ranged from ‘at birth’ to 81 yrs, with equal distribution of cases between males and females. The most common site affected was buccal mucosa followed by buccal vestibule/sulcus, palate, floor of the mouth and tongue. Clinical presentation in most of the cases was an asymptomatic swelling and rest of them presented with facial asymmetry, except for one case which had an accompanying feature of congenital cleft palate. Our case was a 38 yr old female patient with a painless swelling in buccal mucosa.

Most of the cases of osteolipoma of oral cavity presented as painless, well circumscribed, nodular masses with soft-hard in consistency (3). In our case, multiple hard nodular structures could be palpated within the soft and fluctuant lesion, which again was not reported earlier.

Different theories are proposed for the osseous changes in lipomas. Some believe that osteolipoma is a benign mesenchymoma and that both adipose and osseous tissue could originate from multipotent undifferentiated mesenchymal cells. Others believe that neoplastic changes could occur in the fat cells and bone is then formed by metaplasia of fibroblasts to osteoblasts (4). Osteoinducting factor released by blood borne monocytes that enter the fatty tissue could be responsible for transformation of fibroblasts into osteoblasts (5).

Local factors such as chronic micro-trauma and compromised blood supply could act as osseous-metaplasia-inducing factors in lipoma. Since the present lesion was located in buccal mucosa, in line of occlusion, we believe that chronic trauma to the lesion from the masticatory forces could be the triggering factor for calcification/ossification process. Moreover, ossification is known to be associated with lipomas of longer duration. The duration of oral osteolipoma ranged from 2 months to 40 years as per the literature review. The present case had a history of 28 yrs duration of lesion in the oral cavity.

Out of the 16 cases of osteolipomas of oral cavity reported in the literature, only 8 case reports documented the radiographic/imaging findings ( Table 1). Only one case reported by Amaral MB *et al.* (6) had radiographic findings similar to our case i.e., multiple dense homogenous radio-opaque structures. Other cases had radiographic presentation of a mass with area of calcification (7), patchy areas of radio-opacity (8); irregular area of radio-opacity, mixed hypodense and hyperdense areas on CT; round area of radio-opacity with irregular pattern of trabeculae (9). Multiple calcifications of oral soft tissues is commonly seen in vascular lesions (phleboliths) and oral cysticercosis. However, phleboliths are concentric layered calcifications seen in long standing vascular lesions and oral cysticercosis may exhibit multiple ‘rice grain’ pattern of calcifications, which were not observed in the present case.

Histologically, adipose tissue is often mixed with trabeculae of bone in osteolipoma, which was clearly evident in the present case.

Treatment of oral osteolipoma is complete surgical excision, which shows no recurrences and prognosis is similar to that of the other lipomas (10). Surgical excision of the lesion was done in the present case with no recurrence after six months of follow-up.

In conclusion, oral osteolipoma is a rare entity, wherein we report a case affecting retro-commissural area. Osteolipoma should be considered as a radiographic differential diagnosis for multiple calcified dense radio-opaque structures involving oral soft tissues.
